# Biodegradation of Crystalline Cellulose Nanofibers by Means of Enzyme Immobilized-Alginate Beads and Microparticles

**DOI:** 10.3390/polym12071522

**Published:** 2020-07-09

**Authors:** Arnaud Kamdem Tamo, Ingo Doench, Aliuska Morales Helguera, Daniel Hoenders, Andreas Walther, Anayancy Osorio Madrazo

**Affiliations:** 1 Institute of Microsystems Engineering IMTEK, Laboratory for Sensors, University of Freiburg, 79110 Freiburg, Germany; arnaud.kamdem@imtek.uni-freiburg.de (A.K.T.); ingo.doench@imtek.uni-freiburg.de (I.D.); 2 Freiburg Materials Research Center FMF, University of Freiburg, 79104 Freiburg, Germany; daniel.hoenders@makro.uni-freiburg.de (D.H.); andreas.walther@makro.uni-freiburg.de (A.W.); 3 Freiburg Center for Interactive Materials and Bioinspired Technologies FIT, University of Freiburg, 79110 Freiburg, Germany; 4 Chemical Bioactive Center CBQ, Molecular Simulation and Drug Design Group, Central University of Las Villas, Santa Clara 54830, Cuba; aliuska@uclv.edu.cu; 5 Institute for Macromolecular Chemistry, University of Freiburg, 79104 Freiburg, Germany

**Keywords:** polymer microparticles, beads, cellulose nanofibers, alginate, enzyme immobilization, biodegradation

## Abstract

Recent advances in nanocellulose technology have revealed the potential of crystalline cellulose nanofibers to reinforce materials which are useful for tissue engineering, among other functions. However, the low biodegradability of nanocellulose can possess some problems in biomedical applications. In this work, alginate particles with encapsulated enzyme cellulase extracted from *Trichoderma reesei* were prepared for the biodegradation of crystalline cellulose nanofibers, which carrier system could be incorporated in tissue engineering biomaterials to degrade the crystalline cellulose nanoreinforcement in situ and on-demand during tissue regeneration. Both alginate beads and microparticles were processed by extrusion-dropping and inkjet-based methods, respectively. Processing parameters like the alginate concentration, concentration of ionic crosslinker Ca^2+^, hardening time, and ionic strength of the medium were varied. The hydrolytic activity of the free and encapsulated enzyme was evaluated for unmodified (CNFs) and TEMPO-oxidized cellulose nanofibers (TOCNFs) in suspension (heterogeneous conditions); in comparison to solubilized cellulose derivatives (homogeneous conditions). The enzymatic activity was evaluated for temperatures between 25–75 °C, pH range from 3.5 to 8.0 and incubation times until 21 d. Encapsulated cellulase in general displayed higher activity compared to the free enzyme over wider temperature and pH ranges and for longer incubation times. A statistical design allowed optimizing the processing parameters for the preparation of enzyme-encapsulated alginate particles presenting the highest enzymatic activity and sphericity. The statistical analysis yielded the optimum particles characteristics and properties by using a formulation of 2% (w/v) alginate, a coagulation bath of 0.2 M CaCl_2_ and a hardening time of 1 h. In homogeneous conditions the highest catalytic activity was obtained at 55 °C and pH 4.8. These temperature and pH values were considered to study the biodegradation of the crystalline cellulose nanofibers in suspension. The encapsulated cellulase preserved its activity for several weeks over that of the free enzyme, which latter considerably decreased and practically showed deactivation after just 10 d. The alginate microparticles with their high surface area-to-volume ratio effectively allowed the controlled release of the encapsulated enzyme and thereby the sustained hydrolysis of the cellulose nanofibers. The relative activity of cellulase encapsulated in the microparticles leveled-off at around 60% after one day and practically remained at that value for three weeks.

## 1. Introduction

The outstanding mechanical properties of crystalline cellulose nanofibers, the high availability of cellulose in nature, and the renewability and proved cytocompatibility of the nanofibers make them an excellent candidate for the reinforcement of biomaterials in tissue engineering applications [1,2,3,4,5,6,7]. Ideal biomaterials for biomedical applications should have good biocompatibility and biodegradability, and in the case of mechanically demanding tissues, they should have high mechanical strength [8,9,10,11,12,13,14,15,16,17]. In tissue engineering, cellulose fiber (CNF) filled composites can be engineered to mimic fiber-reinforced biological tissues [13,14]. In addition to excellent mechanical properties, crystalline nanofibers of polysaccharides combine important features such as good barrier properties, hydrophilicity, biocompatibility, etc. [18,19,20,21]. Two main types of nanofiber families are defined as cellulose nanowhiskers (CNWs) or microfibrillated cellulose (MFC). The first ones consist of rod-like monocrystals of high stiffness (114–140 GPa), [22,23,24,25] generally obtained by acid hydrolysis of native cellulose substrates [16,21,26]. Alternatively, MFC or nanofibrillated cellulose results from ‘peeling’ off the native cellulose microfibrils into a hairy network of nanofibrils, which are processed by mechanical action in combination with a chemical or biological modification [21,27,28,29]. The excellent mechanical performance of the nanofibers derives from the highly crystalline structure of native cellulose and the high aspect ratio that can be achieved after nanofiber processing. To make use of the excellent mechanical properties and biocompatibility of cellulose nanofibers, it would be advantageous to achieve their biodegradability in vivo, since biomaterial functionalities can depend on the length of time for which they are needed to remain in the body [30,31]. The integration of enzyme carrier systems within cellulose nanofiber-filled tissue engineering biomaterials could be the solution to enable the controlled biodegradation of these crystalline nanofibers over the long term, as required for the specific tissue repairing/regeneration. In biomedicine, CNFs have been used in the reinforcement of tissue engineered biomaterials [13,32,33], protein immobilization [34], drug delivery systems [35], etc. In hydrogel biomaterials, the use of CNFs is promising. In addition to mechanical performance, the CNFs present high water retention, transparency, and biocompatibility [32]. In addition, the CNFs can be oriented within hydrogels by uniaxial stretching under controlled humidity to achieve anisotropic functional biomaterials [16]. For example, injectable formulations and hydrogels filled with CNFs were used respectively as biomaterial for intervertebral disc nucleosupplementation and as a matrix for the culture of fibroblast cells, revealing the potential of CNF reinforced materials for tissue engineering [13,14]. Cellulose-nanofiber reinforced chitosan biomaterials were applied in the repairing of the mechanically-demanding tissue intervertebral disc with promising results achieved in ex vivo investigations with pig animal models [14]. However, the low biodegradability of nanocellulose can possess some problems in biomedical applications. Because of its highly crystalline packed structure and its β(1→4)-linked polysaccharide structure, the CNFs are highly resistant to degradation in the human body. Besides, neither stomach acid nor the hydrolyzing enzymes of the human gastro-intestinal tract are capable to significantly degrade cellulose [36]. To overcome this problem, cellulase enzymes could be integrated within biomaterials to hydrolyze β(1→4) linkages of cellulose [37,38,39,40,41,42,43,44,45,46,47]. A strategy would be the incorporation of enzyme delivery systems within the cellulose-nanofiber biomaterials to allow sustained and on-demand degradation of nanocellulose while the biomaterial plays a bioactive and mechanical role during tissue regeneration.

Cellulase enzymes can be isolated from cellulolytic fungi such as *Aspergillus*, *Humicola*, *Penicillium*, and *Trichoderma*. They are composed of three enzymes that act synergistically in the hydrolysis of cellulose into soluble sugars [48,49,50]. The widespread practical applications of cellulase are limited because of its hydrophilic nature and low stability in various pH and temperature ranges. These challenges could be overcome by sustained release of cellulase into the cellulose-based substrates. Immobilization or encapsulation of cellulase can protect the proteins from interactions with the substrate components and environmental changes, thereby retaining cellulase stability and functionality [48,51]. Polysaccharide materials play an important role in the encapsulation field. Their main functions are the successful encapsulation, transportation, and controlled release of the carrier content to the external environment [52,53,54]. Several natural polymers have been used to enclose and protect bioactive molecules, due to their design versatility and the opportunity to insert various functionalities in the structure itself [55]. Among these biopolymers, alginate has unique properties which have enabled its use as a matrix for the entrapment and delivery of a large variety of proteins and cells. Alginate is a biocompatible and biodegradable linear copolymer, consisting of (1,4)-linked β-D mannuronate (M) and its C-5 epimer α-L-guluronate (G) residues [56]. The monomers can appear in homopolymeric blocks of G- (G-blocks) and M-residues (M-blocks) respectively, or blocks of alternating M- and G-residues. Depending on the source, alginates differ both in the M/G ratio, distribution and length of each block [53,57]. A relatively mild gelation process of alginate in a gelling bath containing multivalent metal ions like Ca^2+^, enables biomolecules and cells to be incorporated into the biocompatible and biodegradable alginate matrix with the retention of the three-dimensional arrangement of the encapsulated molecule [58]. In a number of studies, alginate has been used in the design and fabrication of biomaterials for tissue engineering, recently including 3D bioprinted scaffolds [59,60].

The aim of this work is the preparation of cellulase-immobilized polymer particles enabling the enzyme controlled release for the hydrolysis of crystalline cellulose nanofibers of the type nanofibrillated cellulose. Furthermore, the cellulase-immobilized particle system should find application in the in situ degradation of the nanocellulose incorporated in biomaterials, while this latter serves as a matrix for tissue regeneration. In this work, cellulase was encapsulated in alginate particles and the enzymatic activity was evaluated on three different cellulose substrates: solubilized carboxymethylcellulose (CMC), non-modified (CNFs), and 2,2,6,6-tetramethylpiperidinyloxy (TEMPO)-oxidized cellulose nanofibers (TOCNFs) in suspension. Comparative studies of cellulose nanofiber biodegradation were performed with cellulase-entrapped in alginate beads and microparticles. Microsized particles should possess advantages over larger particles in drug-controlled release and other biomedical applications. These latter were manufactured to have a more uniform size and shape with high surface area-to-volume ratio. Different parameters were varied in the processing of the cellulase-encapsulated alginate particles like the concentration of alginate and ionic crosslinker Ca^2+^, addition of NaCl to change the medium ionic strength, and hardening time. A statistical design enabled us to report the optimum conditions for the processing of spherical particles presenting the highest enzymatic activity. The activity and stability of the immobilized cellulase were compared to those of the free enzyme. The comparative kinetics behavior of the enzymatic degradation of the different cellulose nanofibers both by using alginate beads and micro particles could be discussed.

## 2. Materials and Methods

### 2.1. Starting Materials—Preparation and Characterization

Sodium alginate (ALG) was purchased from Sigma Aldrich (Steinheim, Germany). Weight- and number-average molecular weights of ALG were determined by size exclusion chromatography (SEC). Alginate aqueous solutions at 0.1% (w/v) were prepared in 0.1 M NaNO_3_ adjusted to pH 7, which was also used as eluent. Then, they were filtered through 0.45 µm pore size membrane (CME, Merck-Millipore, Burlington, MA, USA). The chromatographic equipment was composed of an IsoChrom LC pump (Spectra-Physics, Andover, MA, USA) connected to PL aquagel-OH MIXED-M and PL aquagel-OH MIXED-H columns (Agilent Technologies, Santa Clara, CA, USA). A multiangle laser light scattering (MALLS) detector DAWN DSP (Wyatt Technology, Toulouse, France) operating at 664.0 nm was coupled on line to a WATERS 410 differential refractometer. The refractive index increment dn/dc was 0.134 mL/g. The size exclusion chromatography (SEC) coupled to MALLS allowed determining a weight (Mw) and a number-average molecular weight (Mn) of alginate of 9.62 × 10^4^ (±0.66%) and 4.55 × 10^4^ (±2.21%) g/mol, respectively; with a polydispersity index (Mw/Mn) of 2.11 (±2.30%), showing a relatively narrow molecular weight distribution (Figure S1, Supplementary Materials). The guluronic (G)/mannuronic acid (M) composition of the sodium alginate was estimated by means of Fourier transform infrared spectroscopy FTIR. KBr pellets containing 1% (w/w) of alginate were prepared to record the FTIR spectrum on a Perkin Elmer instrument (Spectrum 65, Germany) in the 600–4000 cm^−1^ range. From the alginate FTIR spectrum (Figure S1, Supplementary Materials), a composition of (79 ± 3)% of G and (21 ± 3)% of M was determined; by using the ratio of absorbances of bands at 1320 and 1290 cm^−1^, and at 1125 and 1030 cm^−1^ as proposed by Filippov and Kohn [61].

Carboxymethylcellulose (CMC) was supplied by Sigma Aldrich (Germany). It is a derivative of cellulose, which is soluble in aqueous buffers and others solvents.

Cellulose nanofibers of the nanofibrillated cellulose type were used:

(i) Non-modified cellulose nanofibers (CNFs): Their suspensions were obtained from bleached pine sulfite dissolving pulp at the Centre Technique du Papier (CTP, Grenoble), by a mechano-enzymatic method adapted from Pääkkö et al. (2007) [28]. The pulp was refined at 4.5% consistency with a 12″ single disk refiner for 25 min. Then, it was incubated at 50 °C for 1 h with a solution of endoglucanase FiberCare R^®^ (Novozyme) at pH 5.0. The digested samples were further refined to obtain a pulp suspension of SR (Schopper-Riegler) number higher than 80 and mean fiber length lower than 300 µm. 2% (w/w) fiber suspensions were processed with an Ariete homogenizer, involving one pass at 1000 bar followed by three passes at 1500 bars.

(ii) TEMPO-oxidized cellulose nanofibers (TOCNFs): They were prepared according to the protocol described by Benitez et al., 2016 [62]. Kraft pulp was suspended in a 0.05 M sodium phosphate buffer at 60 °C, containing TEMPO and sodium chlorite. Then, a sodium hypochlorite solution was added to initiate the oxidation. The mixture was maintained at pH 6.6–7.0 and vigorously stirred for 1 h. The oxidation was quenched by adding ethanol at the end of the reaction. The product was washed with deionized water. To obtain TEMPO-oxidized cellulose nanofibrils, the suspension of the TEMPO-oxidized pulp was homogenized in a LM10 Microfluidizer applying four shear cycles (2 × 1400 bar, 2 × 1000 bar). The obtained cellulose nanofibers contained 0.44 mmol/g of carboxylic groups.

The enzyme cellulase Celluclast 1.5 L from the fungus *Trichoderma reesei* was purchased from Novozyme (Bagsvaerd, Denmark). Celluclast 1.5 L had a protein content of 124 mg/mL and activity of 700 EGU/g.

### 2.2. Preparation of Cellulase-Encapsulated Alginate Particles

(i) Beads: Alginate was dissolved at a given concentration (2%, 3%, or 5% (w/v)) in Milli-Q water, by mechanical stirring for 24 h at 60 °C. The obtained viscous solution was filtered through 0.8 µm pore size membrane (CME, Merck-Millipore). Then, alginate beads were prepared by extruding the alginate solution by means of a dispenser PerformusTM I (Nordson EFD, East Providence, RI, USA). It allowed the pumping, under controlled dry air pressure (0.3 bar) of the solution contained in a syringe cartridge, through a needle of inner diameter 200 µm. The dropping distance between the needle tip and the surface of the CaCl_2_ coagulation bath was adjusted to 10 cm. Different CaCl_2_ concentrations (0.2 M or 0.4 mol/L) were considered. In some experiments, 0.8 mol/L NaCl was added to increase the medium ionic strength and thereby promote the formation of homogeneous spherical beads. These later were allowed to harden for 1 or 3 h in the CaCl_2_ crosslinking bath. Finally, the beads were collected, washed several times with Milli-Q water and directly stored as hydrogel beads in pH 4.8 acetate buffer at 4 °C, or freeze-dried to obtain dry particles. To prepare cellulase-encapsulated alginate beads, Celluclast 1.5 L from *Trichoderma reesei* was mixed with the alginate solution and left gently stirring for 1 h at 4 °C. A relation of 1 mg of enzyme protein to 290 mg of alginate was used. Then, same dispensing procedure as above was followed to obtain cellulase-entrapped alginate beads.

(ii) Microparticles: An inkjet-based method was used to prepare alginate microparticles. A 4% (w/v) alginate viscous solution (bioink) was loaded into a cartridge, which was set to a temperature-controlled nozzle system, constituting a printhead of a 3D bioprinter (3D Discovery, RegenHU Ltd., Villaz-St-Pierre, Switzerland). Preliminary works were performed with lowest alginate concentrations (2%, 3%, and 3.5% (w/v)), but distinct spherical particles of homogeneous size distribution were not achieved. The alginate microparticles were ‘printed’ into a gelling bath containing 0.2 M CaCl_2_ and 0.2 M NaCl, by applying a pressure of 0.7 MPa, a dosing distance 0.03 mm and a valve opening and closing time of 250 and 300 µs, respectively. They were allowed to harden for 1 h while gently magnetically stirring. Finally, the microparticles were washed several times with Milli-Q water and freeze-dried. To achieve cellulase-encapsulation in the microparticles, in the inkjet ‘printing’ procedure, alginate solutions containing the same cellulase/alginate ratio as above (1/290 mg/mg) were considered.

### 2.3. Cellulose Enzymatic Degradation—Cellulase Activity Assay

The cellulose degradation was carried out by enzymatic hydrolysis with cellulase immobilized in the alginate particles or as free enzyme. Measures of 20 mg of cellulase-encapsulated alginate particles or the corresponding amount of free enzyme dissolved in few µL of acetate buffer at a given pH were incubated with the solubilized carboxymethylcellulose (CMC) or with the cellulose nanofibers in suspension. The cellulose hydrolyses were performed at varied temperatures between 25 and 75 °C, pH values between 3.5 to 8.0, and incubation times of 21 days. On a practical level of understanding the effect of environmental parameters on enzymatic activity, previous studies have called for the caution of the common practices of using soluble substrates [63], like the CMC—a carboxylated derivative of cellulose as the here used cellulose nanofibers—for the general characterization of pH and temperature effects on cellulase activity. For the CMC degradation studies in solution (homogeneous conditions), 20 mg of cellulase-encapsulated alginate particles were added to 900 µL of a 1% (w/v) solution of CMC in acetate buffer at a given pH. In heterogeneous conditions, 20 mg of cellulase-encapsulated alginate particles were added to a 0.2% (w/v) suspension of cellulose nanofibers. The enzymatic hydrolysis was stopped by adding 2 mL of 0.044 M dinitrosalicylic acid (DNS) solution. The product was heated for 5 min upon which a red-brown color developed, due to the reduction of the DNS by the sugar reducing ends into 3-amino-5-nitrosalicylic acid, which can be quantified by UV spectrophotometry at 540 nm, wavelength of maximum absorbance. The activity of the free and immobilized cellulase was determined by measuring the amount of sugar reducing ends produced after enzymatic hydrolysis of the cellulose substrate. Thus, the sugar reducing ends concentration was quantified by the DNS method at a wavelength of 540 nm, using an UV-visible spectrometer. The unit of cellulase catalytic activity (IU) is defined as the amount of cellulase that hydrolyzes the cellulose substrate to produce 1 μmol glucose per minute. Each experiment was performed in triplicates. The catalytic activity of the enzyme was determined and the effect of pH, temperature, and incubation time on the enzyme activity could be investigated.

### 2.4. Microscopical Observations

(i) Atomic force microscopy (AFM): The morphology of the cellulose nanofibers was characterized by atomic force microscopy (AFM). A droplet of a 0.001% nanofiber suspension was placed on a freshly cleaved mica surface. After sample drying, the observation was performed in tapping mode with an AFM (NanoScope V Controller and NanoScope 9.1 Software, Bruker Corporation, Santa Barbara, CA, USA) equipped with a tube scanner from Veeco Digital Instruments (Santa Barbara, CA, USA), using silicon tips (PPP-NCH, Nanosensors, Sindelfingen, Germany) with resonance frequency and spring constant of 360 kHz and 50 N·m^−1^, respectively. Height and phase images were analyzed with NanoScope Analysis 1.5 software.

(ii) Optical microscopy: The dimensions and morphology of the alginate particles, both as hydrogel and dry state, were analyzed by light optical microscopy. To this end, a digital camera (Moticam 2.0 MP, Hong Kong, China) coupled to a binocular optical microscope (VWR International GmbH, Bruchsal, Germany) was used. The particle micrographs were analyzed with the software ImageJ 1.52u. Concerning bead shape analysis, the sphericity factor SF was determined by using the Equation (1). This factor varies from 0 for a perfect sphere up to unity for an elongated object [64,65,66].

SF = (*d*_max_ − *d*_per_)/(*d*_max_ + *d*_per_) (1)

where *d*_max_ is the maximum diameter passing through a bead centroid; and *d*_per_ is the diameter perpendicular to *d*_max_.

(iii) Fluorescence microscopy: Fluorescence microscopy allowed observing the local distribution of the enzyme within the alginate particles. The cellulase was stained with fluorescein isothiocyante (FITC). In dark conditions, 700 µg of FITC reactive dye were dissolved in dimethyl sulfoxide (DMSO) to achieve a concentration of 10 mg/mL FITC. 2 mg of cellulase were added to 1 mL of sodium bicarbonate buffer (pH 9.0) and the obtained solution was stored at 4 °C for 1 d. The FTIC solution from above was added into the flask containing the enzyme solution and the mixture was incubated for 8 h at 4 °C in darkness. Dialysis was used to remove the unconjugated FITC. The inner content of the dialysis tubing containing the FITC-labeled cellulase was collected. The stained enzyme was used to prepare alginate particles with encapsulated fluorescent-labeled cellulase, following the above described procedure to prepare the cellulase-encapsulated alginate particles. The appearance of the FITC-labelled cellulase alginate beads after immersed in PBS solution (pH 7.4) for different times (0, 1, 2, 6, 24, 96, 192, 264, and 336 h) was followed by fluorescence microscopy. The fluorescent beads were imaged by using a confocal laser scanning microscope (Leica TCS SPE, Wetzlar, Germany) equipped with detectors and filter sets for monitoring fluorescence.

(iv) Scanning electron microscopy (SEM): The dimensions and surface morphology of the beads and alginate microparticles was also investigated by scanning electron microscope (Amray 1610 Turbo, Anray Inc., Bedford, MA, USA) at an accelerating voltage of 15 kV. For SEM measurements, the freeze-dried particles were deposited on conductive carbon tabs and coated with gold under vacuum using a sputter-coater.

### 2.5. X-ray Diffraction

X-ray diffraction (XRD) patterns of the cellulase-encapsulated alginate particles, the sodium alginate, and the cellulose nanofibers both CNF and TOCNFs after different hydrolysis times were determined using a diffractometer AXS D8 (Bruker Karlsruhe, Germany), operating at 40 kV and 40 mA with the Cu Kα radiation (λ = 1.542 Å). The diffraction angle 2θ varied between 5 and 120° by steps of 0.04° for the starting materials: alginate, CNFs, TOCNFs, and for the obtained cellulase-encapsulated alginate particles; and between 5 and 70° by steps of 0.02° for the CNFs and TOCNFs obtained after different times of enzymatic degradation.

### 2.6. Thermogravimetric Analysis

Thermogravimetric analysis TGA of the cellulase-encapsulated alginate particles and starting alginate was performed with a STA 409 C equipment (Netzsch Gerätebau GmbH, Selb, Germany). Approximatively 10 mg of the dried samples were weighed in a platinum pan and heated from room temperature up to 700 °C, with a heating rate of 10 °C/min under nitrogen atmosphere.

## 3. Results and Discussion

### 3.1. Particles Morphology and Dimensions

**Figure 1:**
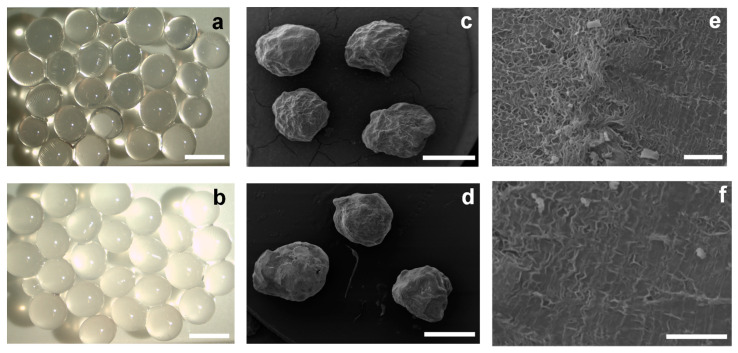
(**a**,**b**) Optical micrographs of hydrogel alginate beads of: (**a**) formulation F1 (Table 1), scale bar: 3 mm; (**b**) formulation F3 (F1 with addition of 0.8 M NaCl, Table 1), scale bar: 3 mm. (**c**–**f**) SEM micrographs of the corresponding dried beads: (**c**,**d**): F1 and F3, respectively, scale bars: 500 μm; (**e**,**f**) SEM micrographs at higher magnification showing the surface morphology of F1, scale bars: 2 μm.

Figure 1 shows optical and SEM micrographs of the alginate beads. The hydrated beads showed good spherical shape with a smooth and homogeneous surface. With the freeze-drying, a shrinkage of the particles occurred and a less spherical shape with a wrinkled and porous surface was distinguished by SEM. The addition of the enzyme cellulase did not appreciably change the shape and appearance of the corresponding alginate particles, indicating that enzyme encapsulation does not significantly change bead morphology [67].

Table 1 shows the mean particle size of the obtained hydrogel and dried beads processed after using different formulations with varied parameters like the concentrations of alginate and CaCl_2_ coagulation bath, addition or not of NaCl, and hardening time (HT). In general, the size of the beads ranged from (2.0 ± 0.4) to (2.5 ± 0.2) mm for the hydrated and (1.1 ± 0.2) to (1.7 ± 0.2) mm for the dried beads. The size and shape of the beads should be influenced by internal and external factors including surface tension of alginate solution and gelation bath, collecting distance, CaCl_2_ concentration, needle diameter, M/G ratio, distribution in alginate chains, and stirring rate of gelation bath [68,69]. When the solution droplet hits and enters in the gelling bath, there are competing forces between the viscous surface tension and impact-drag forces to maintain the droplet shape [65]. The sphericity factor (SF) increased with the increase of alginate concentration (c(ALG), Table 1). Thus, the increase of the latter led to less spherical particles, likely due to the higher viscosity of the alginate solution with higher force per unit of area required to dispense it through the extrusion needle.

### 3.2. Activity of Encapsulated Cellulase towards Hydrolysis of Cellulose Substrate in Solution

#### 3.2.1. Effect of Temperature

**Figure 2:**
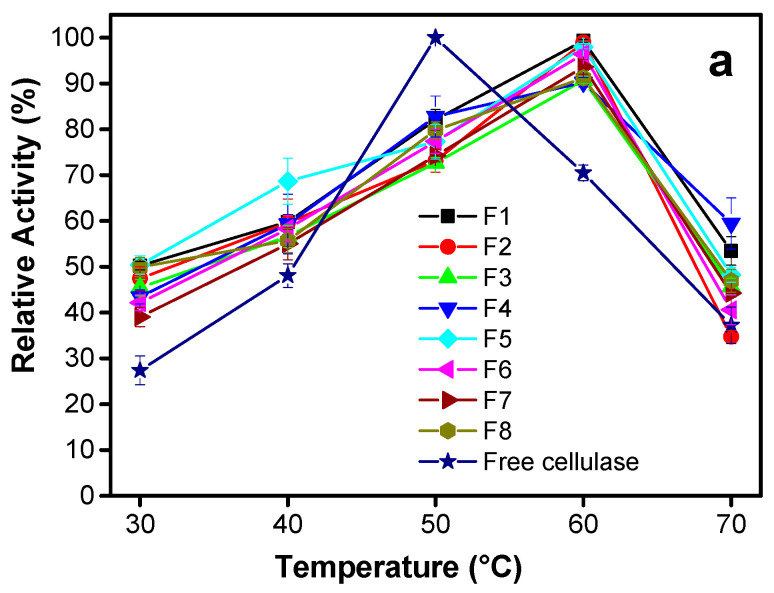

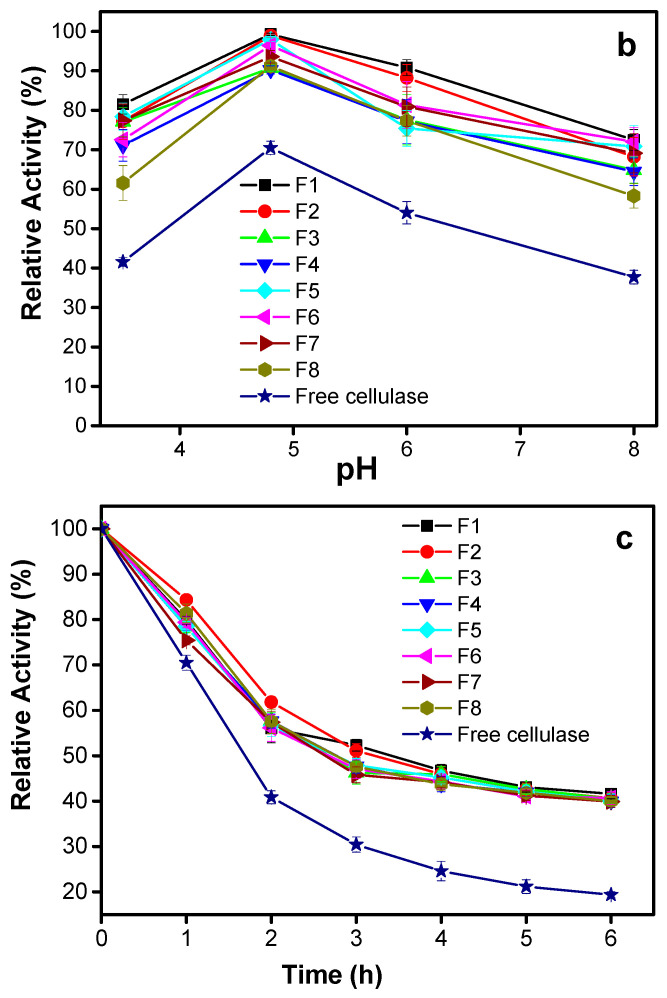
Relative activity of cellulase encapsulated in alginate beads prepared by using 2% (w/v) alginate formulations (F1–F8, Table 1) in comparison to that of the free enzyme, showing the degradation of carboxymethylcellulose at: (**a**) different temperatures after 1 h at pH 4.8; (**b**) different pH after 1 h at 60 °C; (**c**) different times at pH 4.8 and 60 °C.

Figure 2 shows the effect of temperature on the activity of immobilized and free cellulase in the biodegradation of cellulose substrate (CMC) in solution, for formulations prepared with 2% (w/v) alginate. The results obtained by using 3% and 5% (w/v) alginate formulations are shown in Supplementary Materials (Figure S2). The studies at varied temperature were performed for 1 h at pH 4.8. The maximum activity was obtained at 60 °C for the immobilized enzyme while at 50 °C for the free enzyme. The immobilized cellulase activity decreased with the increase of c(ALG), as the decrease of particle porosity should slow the diffusion of the enzyme. In comparison to free enzyme, the immobilization enabled extending the spectrum of effective utilization of the enzyme at higher temperatures (Figure 2, and Figure S2 in Supporting Material), indicating that the immobilization improves the thermal stability of the enzyme. The cellulases from *Trichoderma reesei* present carbohydrate binding moieties which might favor interaction of the enzyme with the alginate matrix [63]. It should contribute to slightly shift the temperature of maximum enzyme stability to higher values when being entrapped in the alginate matrix. Nevertheless, at the position of maximum activity temperature, lower enzyme activity is observed in comparison to the free enzyme (Figure 2), and this effect is enhanced when the concentration of alginate increases (Figure S2 in Supporting Material), which seems to confirm the role of the interaction enzyme-carbohydrate alginate matrix on enzyme thermal stability. The general trend of increase of the activity of the immobilized enzyme at the temperature range 30–60 °C could be due to reduced conformational flexibility of the enzyme because of encapsulation. Like most chemical reactions, the rate of an enzyme-catalyzed reaction increases as the temperature increases. Nevertheless, towards the highest temperatures the enzyme stability can be adversely affected, the reaction rate increases with temperature to a maximum level, then it abruptly declines with further increase of temperature [69].

#### 3.2.2. Effect of pH

Figure 2 and Figure S2 in Supplementary Materials show that the maximum of activity of the entrapped enzyme was recorded at pH 4.8 as for the free cellulase. The pKa of guluronic acid (G) and mannuronic acid (M) in 0.1 M NaCl are reported to be 3.65 and 3.38 respectively [70]. Thus, the G-M copolymer (alginate) is insoluble or in the collapse state at pH values below the pKa, while soluble or in the swollen state at pH values higher than pKa. It has been reported that cellulases enzymes have optimal stability between pH 4 and 5. In addition, binding to a substrate promotes enzyme stability. Binding of the enzyme to alginate in a pH at which the alginate is in its swollen state (slightly above its pKa)—i.e., not dissolved and not in its collapse/insolubility state—seems to be the perfect combination to match with the pH of optimal enzyme stability. Finally, it has been also observed that the strong loss in activity at lower pHs around pH 2–3 at 60–70 °C are most likely caused by enzyme instability, as thermal stability analyses showed that the Tm of the enzyme under these conditions hovers around or below the usually reported temperature (Tm is 50–60 °C) [63]. Then, the reduction in activity at lower temperatures 20–40 °C (supposed to be below Tm) and pH 5–6 is probably not related to instability, as Tm is much higher under these conditions. Thus, some investigations have inferred possible interference from enzymatic activity inhibition due to the degradation product (i.e., cellobiose). The activity of entrapped cellulase was higher than that of the free enzyme in the whole studied pH range; and this was significant at the lowest c(ALG). Through the encapsulation, the multi-point enzyme binding mechanism limits undesired conformation against environment changes which seems to improve stability of the encapsulated cellulase in a broader pH range [71]. It is reported that enzyme immobilization within hydrogel carriers like alginate, poly(hydroxyethylmethacrylate–*co*–glycidylmethacrylate), gelatin and polyacrylamide provides high stability due to the protective microenvironment of the gel matrix [72,73,74,75].

#### 3.2.3. Effect of Incubation Time

The stability of entrapped cellulase was initially investigated along 6 h incubation at pH 4.8 and at 60 °C towards degradation of solubilized cellulose substrate. The relative activity of the cellulase encapsulated in the alginate particles was 44.9%, 42.1%, and 38.4% after 6 h for alginate capsules prepared with alginate 2% (Figure 2), 3%, and 5% (w/v) (Figure S2 in Supplementary Materials) respectively; whereas the free cellulase only presented 19.4% of relative activity. This suggests a screen function of the alginate support, which protects enzyme structure against denaturation.

#### 3.2.4. Optimization for High Catalytic Activity of the Encapsulated Cellulase

To determine the most significant parameters affecting particle sphericity and relative activity of immobilized cellulase after 1 h incubation at the obtained pH and temperature of maximum activity (pH 4.8; 60 °C), the four independent variables: concentrations of alginate, CaCl_2_, NaCl, and hardening time (HT) were considered in a statistical analysis. As mentioned above, the c(ALG) was varied at three levels, while that of CaCl_2_ and NaCl, and the HT were varied at two levels. A 2^3^∙3 full factorial design was used. Table 1 shows the values representing the levels for each studied factor and the 24 formulations considered in the experimental design. The run order of the experiments was randomized in order to prevent systematic errors. The results shown in Table 1 were evaluated with Analysis of Variance (ANOVA) using the software STATISTICA 10.0 (StatSoft Inc: Tulsa, OK, USA, 2011). The significance of the effects was verified with Fisher’s statistical test using 0.05 as the significance level.

ANOVA analyses of the four factors showed that c(ALG) and c(NaCl) were the most significant variables (*p* < 0.05) influencing on both particle sphericity factor (SF) and relative activity of immobilized cellulase. The interaction of these two factors and in a less extension that between the c(CaCl_2_) and the hardening time (HT) have significant influence on the sphericity factor (SF). This is described in more detail in the following section.

#### 3.2.5. Multiple Response Optimization

For the simultaneous consideration of multiple responses, we proposed a fitting response surface model for the particle sphericity SF and the encapsulated cellulase relative activity. The processing conditions were set to optimize those responses, or at least to keep them in a desired range. A second-order polynomial model (Equation (2)) including quadratic (only for the three-level factor) and linear interactions was used to calculate the predicted response.

y = b0 + b1*x1 +…+ bk*xk + b12*x1*x2 + b13*x1 *x3 +…+ bk−1,k*xk−1*xk + b11*x1^2^ +…+ bkk*xk^2^ (2)

where y is the predicted response (SF or relative activity), b0 is the intercept of the model and b1, … bkk are the regression coefficients of the main linear effects for the factors (x1, …, xk), their interactions (x1*x2, x1*x3, …, xk−1*xk), and their quadratic components (x12, …, xk2).

The software STATISTICA 10.0 (StatSoft Inc: Tulsa, OK, USA) was used in all calculations and a significance level of 0.05. Two regression models depicting the relative activity and the SF were obtained. Equations (3) and (4) represent the final empirical models for relative activity and SF, respectively.

(3) $$\begin{array}{lll} Relative\;activity & \\ = & 157.64-36.70c\left(ALG\right)+4.47c^{2}\left(ALG\right)+1.19c\left(CaCl_{2}\right) & \\ - & 18.50c\left(NaCl\right)-1.55HT-7.22c\left(ALG\right)\cdot c\left(CaCl_{2}\right)+2.20c\left(ALG\right) & \\ \cdot & c\left(NaCl\right)+0.16c\left(ALG\right)\cdot HT+16.62c(CaCl_{2})\cdot c\left(NaCl\right) & \\ + & 3.17c\left(CaCl_{2}\right)\cdot HT+1.07c\left(NaCl\right)\cdot HT;\;R^{2}=0.969\;\mathrm{and}\;{R^{2}}_{\mathrm{adj}} & \\ = & 0.940 \end{array}$$

(4) $$SF=-0.08+0.04c\left(ALG\right)-2.91\cdot 10^{-3}c^{2}\left(ALG\right)+0.05c(CaCl_{2})-0.03c\left(NaCl\right)+\;0.01HT-3.57\cdot 10^{-3}c\left(ALG\right)\cdot c(CaCl_{2})+0.01c\left(ALG\right)\cdot c\left(NaCl\right)-5.36\cdot \;10^{-4}c\left(ALG\right)\cdot HT+0.03c\left(CaCl_{2}\right)\cdot c\left(NaCl\right)-0.04c\left(CaCl_{2}\right)\cdot HT+3.12\cdot \;10^{-3}c\left(NaCl\right)\cdot HT;R^{2}=0.977\;\mathrm{and}\;{R^{2}}_{\mathrm{adj}}=0.957$$

The quality of the models could be verified by observing the determination coefficient R^2^. In both equations the R^2^ was close to one and to the adjusted R^2^ (R^2^_adj_). The significance of linear interaction and quadratic model terms was determined using Fisher’s statistical test and *p*-value, as displayed in ANOVA analyses in Tables S1 and S2 of Supplementary Materials.

The c(ALG) and their quadratic term, the c(CaCl_2_), the c(NaCl) and the interaction between c(ALG) and c(NaCl) are the significant model terms for the enzyme relative activity (Table S1 in Supplementary Materials). Concerning the SF model, the c(ALG), the c(NaCl) as well as the interaction terms c(ALG)*c(NaCl) and c(CaCl_2_)*HT are significant model terms (Table S2 in Supplementary Materials). The rest of the terms are negligible for both responses. The factor showing the most significant influence on the relative activity and the SF is the concentration of alginate, displaying the highest F-values of 303.39 and 477.92, respectively (Table S1 and S2 in Supplementary Materials).

According to Equation (3), the c(ALG) has a negative effect on the relative activity, indicating that lower alginate concentration leads to higher enzyme activity. For SF, the c(ALG) has a positive effect (Equation (4)) but it means that better particle sphericity was achieved for lower alginate concentration [64,65,66]. For medium molecular weight alginate, i.e., 96200 g/mol as used in this work, as the alginate concentration increases the solution density slightly increases but the zero shear rate viscosity is expected to suddenly increase [65]. Due to the surface tension effect, the shape of a falling drop evolved from tear-drop shaped to egg-shaped before it eventually became spherical. Thus, a decreasing of the sphericity factor is observed along those dripping stages. If a tear-shaped liquid drop is collected, the liquid drop shape could be partially preserved during impact with the gelling bath. This could result in a bead with a distinct tail. This effect, namely decrease of sphericity, is magnified when the liquid drop is produced from the highest alginate concentration presenting increased viscosity. A negative effect was also observed for the c(NaCl), indicating that the highest activity was obtained without addition of NaCl. It is worth noticing that the Na^+^ ions (monovalent) might compete with the Ca^2+^ (divalent) to link to alginate chains, which could contribute to destabilization of the so called egg-box structure of Ca^2+^-crosslinked alginate. Thus, the Na^+^ could contribute to a partial disruption of the Ca-alginate network constituting the particles. This effect diminishes the protective role of these latter for the enzyme, as the alginate particles serve as support for enzyme sustained delivery and activity preservation.

**Figure 3:**
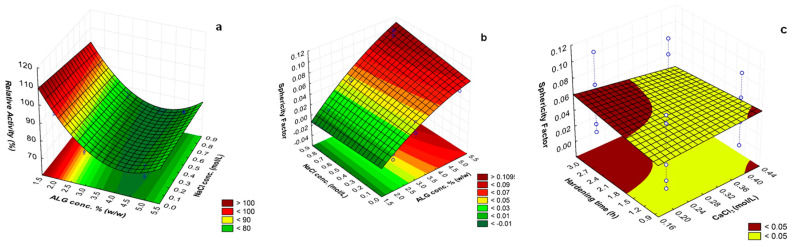
Response surface and contour plots of the combined effects of: concentration of ALG and NaCl on (**a**) the encapsulated cellulase relative activity and (**b**) the alginate particle sphericity factor (SF) when the other two factors c(CaCl_2_) and hardening time (HT) were at center points; (**c**) concentration of CaCl_2_ and HT on the alginate particle sphericity factor (SF) when the other two factors c(ALG) and c(NaCl) were at center points.

The response surface and contour plots of significant combined effects obtained for each response are shown in Figure 3. Figure 3a displays the 3D response surface and contour plots for the combined effects of c(ALG) and c(NaCl) on the immobilized cellulase relative activity, when the other variables were at center points (c(CaCl_2_): 0.3 mol/L; hardening time: 2 h). The enzyme activity significantly increased when the concentration of alginate and NaCl were the lowest. Figure 3b shows the three-dimensional response surface of the combined effects of c(ALG) and c(NaCl) on the SF. Figure 3c shows the combined effects of c(CaCl_2_) and hardening time (HT) on the SF when the other variables were at center points (c(ALG): 3.33 % (w/v) and c(NaCl): 0.4 mol/L). Better sphericity (low SF) was obtained with the decrease of c(ALG), c(NaCl), and c(CaCl_2_), and by shortening the hardening time.

Finally, as both responses are important for the obtaining of particles for sustained release of active cellulase, the desirability function was used to optimize the two responses simultaneously. This function is based on a numerical interval that defines the desirability of the analyst in relation to the optimal condition of the process. The optimization using the desirability function yielded as optimum processing parameters to achieve the spherical particles with the highest enzyme catalytic activity: c(ALG) = 2% (w/v), c(CaCl_2_) = 0.2 mol/L, c(NaCl) = 0 and 1 h hardening time. These optimum conditions corresponded to those used in the formulation F1 (Table 1). Thus, these parameters were considered in the processing of the alginate particles to be used in the studies of degradation of crystalline cellulose nanofibers in suspension (heterogeneous conditions).

### 3.3. Activity of Cellulase Encapsulated in Alginate Beads towards Degradation of Cellulose Nanofibers

**Figure 4:**
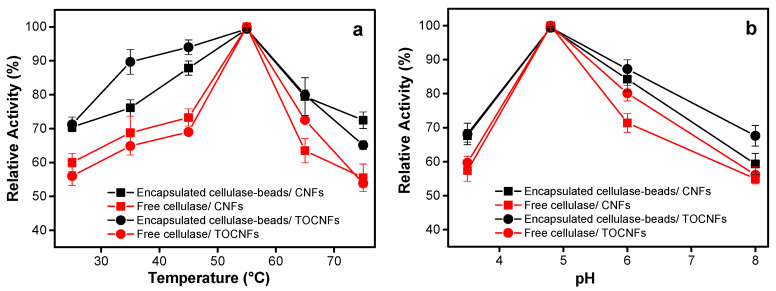
Relative activity of entrapped cellulase in alginate beads of optimized formulation F1, Table 1 (2% (w/v) alginate, 0.2 M CaCl_2_, 1 h hardening time) containing 0.2 M of NaCl, in comparison to free enzyme; showing the degradation of two different cellulose nanofiber samples (CNFs and TOCNFs) after 1 h: (**a**) at different temperatures at pH 4.8; (**b**) at different pHs at 55 °C.

Cellulose nanofibers enzymatic degradation studies in suspension were performed with cellulase-encapsulated alginate beads processed as in the formulation F1 (Table 1) with the small modification of adding 0.2 M NaCl to approach the saline physiological conditions. The cellulase containing beads were incubated with the cellulose nanofibers for 1 h at different temperatures, in order to study the effect of temperature on the enzyme activity in heterogeneous catalysis of semicrystalline substrates. For both nanofiber types (CNFs and TOCNFs), the enzyme activity gradually increases until achieving temperature 55 °C (Figure 4a). Then, the enzymatic activity decreases. The enzyme encapsulation significantly improved the enzyme thermal stability in the studied temperature range. Figure 4b illustrates the activity of the immobilized cellulase at different pHs during the CNFs and TOCNFs degradation in suspension, with activity of the immobilized cellulase being slightly higher than that of the free enzyme and maximum value achieved at pH 4.8.

#### Storage Stability of Cellulase

**Figure 5:**
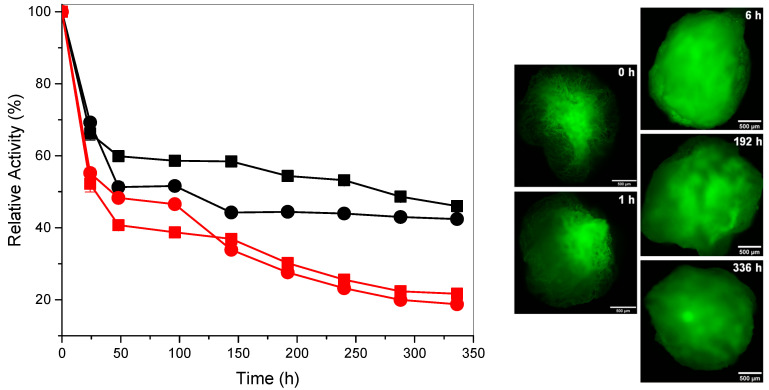
(**Left**) Storage stability of cellulase up to 336 h (14 d) at pH 4.8 and 55 °C, showing the degradation of cellulose nanofibers CNFs (-■-) and TOCNFs (-●-) by the enzyme immobilized in the alginate beads; in comparison to the free enzyme (CNFs (-■-) and TOCNFs (-●-)). (**Right**) Fluorescence micrographs of the alginate particles containing FITC-labelled cellulase, which show the local distribution of the enzyme after different times in a PBS buffer. Scale bars: 500 µm.

Figure 5 shows the activity of entrapped and free cellulase in the time course of degradation of CNFs and TOCNFs at pH 4.8 and 55 °C for which condition maximum activities were obtained above. The decay of the enzyme activity during the long-term degradation of CNFs and TOCNFs was significantly decreased by encapsulating the enzyme in the alginate beads. The enzymatic activity practically achieved a plateau at 48 h and it only started to slightly decrease after 144 h (6 d), with high enzymatic activities of around 50% determined even after 336 h (14 d) and a plateau achieved in the case of TOCNFs nanofibers (Figure 5). In contrast, the free enzyme continuously decreases its activity, reaching values of around 20% after 336 h (14 d).

Fluorescence micrographs in Figure 5 show the local distribution of the FITC labeled-cellulase for different times. At the beginning, the enzyme is mainly located at the core of the particle. With the time, it is more evenly distributed in the whole particle which should be related to its diffusion throughout the particle polymer matrix. Then, the fluorescence intensity decreases for longer times which supports a sustained diffusion of the enzyme throughout the alginate matrix into the surrounding. The enzymatic degradation could be explained due to an enzyme diffusion-based mechanism, allowing the sustained degradation of the cellulose nanofibrils suspended in the alginate particle surrounding.

### 3.4. Cellulase-Immobilized Alginate Microparticles

#### 3.4.1. Microstructure, Dimensions and Properties

**Figure 6:**
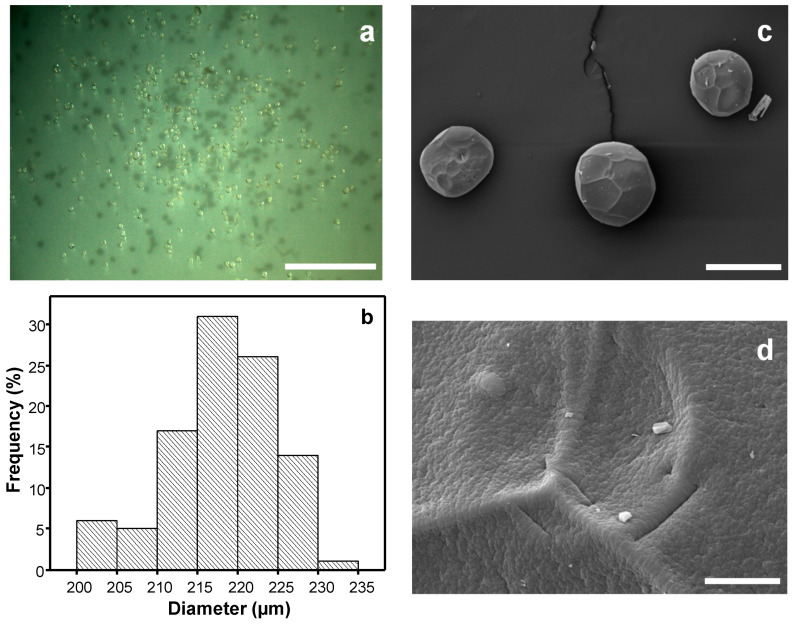
(**a**) Optical micrograph and (**b**) size distribution (mean width: 217.49 ± 6.34 µm) of alginate microparticles obtained by drop-on-demand inkjet technology (4% (w/v) alginate, 0.2 M CaCl_2_ bath with 0.2 M NaCl, 1 h hardening time). (**c**,**d**) SEM micrographs of the alginate microparticles at different magnifications. Scale bars: (**a**) 2 mm, (**c**) 200 μm, (**d**) 8 μm.

Figure 6 shows optical and electron micrographs of the alginate microparticles prepared by the drop-on-demand inkjet technology. They exhibited a homogenous size distribution and shape with a smooth surface. Microparticles with a mean size of 217.49 ± 6.34 µm and a sphericity factor of 0.059 were obtained by dropping a 4% (w/v) alginate solution into a 0.2 M CaCl_2_ bath containing 0.2 M NaCl and leaving hardening the particles for 1 h.

The X-ray diffraction and thermogravimetric analyses of the cellulase/alginate microparticles in comparison to those of the beads are provided in Supplementary Materials (Figure S3). The rapid ionic crosslinking of the alginate microdrops does not seem to give enough time for high molecular ordering of the alginate matrix, as revealed in the X-ray scattering patterns showing a less crystalline microstructure in the microparticles respect to the beads (Figure S3 in Supplementary Materials). It also seems to influence the particle thermal properties (Figure S3 in Supplementary Materials) and the formation of a more dense and homogeneous surface of the microparticles as observed by SEM (Figure 6).

#### 3.4.2. Catalytic Activity of Cellulase Encapsulated in Alginate Microparticles towards Cellulose Nanofibers Degradation

**Figure 7:**
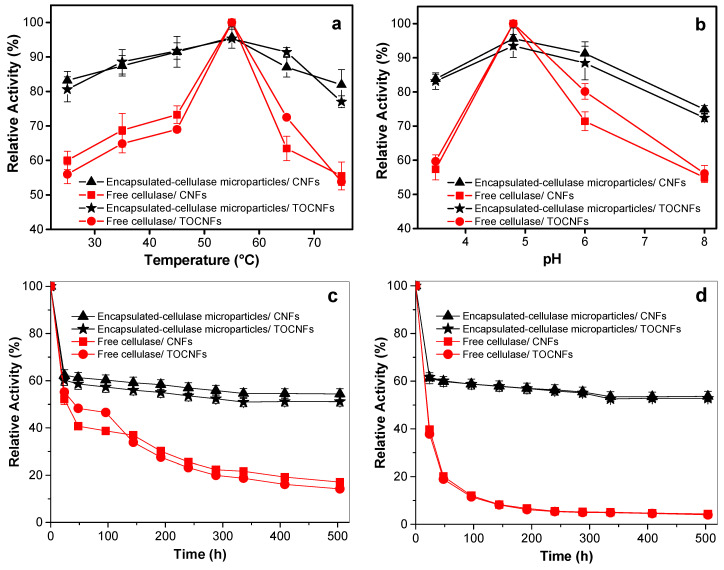
Relative activity of the cellulase immobilized in the alginate microparticles (black curves) and the free enzyme (red curves) for the degradation of CNFs and TOCNFs cellulose nanofibers at: (**a**) different temperatures after 1 h at pH 4.8; (**b**) different pHs after 1 h at 55 °C; different incubation times at: (**c**) conditions of maximum activity of the enzyme (pH 4.8, T = 55 °C, (**d**) conditions resembling more the physiological environment (pH 6.5, T = 37 °C).

The catalytic activity of the cellulase-entrapped microcarriers was tested towards degradation of cellulose nanofibers in suspension. The effects of pH, temperature and incubation time on the activities of entrapped and free cellulase were also investigated (Figure 7). The storage stability of entrapped cellulase was assessed by incubating the cellulase-entrapped alginate microparticles with the two types of cellulose nanofibers (CNFs and TOCNFs) until 504 h (21 d) in two different conditions: optimum conditions as above (pH 4.8 and 55 °C, Figure 7c) and those approaching more physiological environments (pH 6.5 and 37 °C, Figure 7d).

The cellulase encapsulated in the alginate microparticles also exhibited their maximum activity at pH 4.8 and temperature 55 °C during the cellulose nanofibers biodegradation (Figure 7a,b). In comparison to the above studies with the beads, it is worth noticing that although the activity maximum was not that distinct for the enzyme encapsulated in the microparticles, this was significantly higher than those of the free enzyme. This effect was significant at pH and temperature differing from those positions of maximum activity. It highlights the advantage of the high surface area-to-volume ratio of the microparticles over that of large beads.

The loss of the activity of the cellulase entrapped in the microparticles already started to be negligible after 3 h. The relative activity remained high and practically achieved a plateau at around 60%. Nevertheless, the free enzyme lost its activity very fast. Notably, in the degradation studies performed at pH 6.5, T = 37 °C this loss suddenly happened at very early stage with only 7% activity observed after 192 h (8 d) for the free enzyme, while the encapsulated cellulose retained a high activity of 60% until at least 504 h (21 d) (Figure 7d).

### 3.5. Kinetics Study of the Biodegradation of Cellulose Nanofibers

In order to quantitatively study the kinetics of the cellulose nanofibers enzymatic hydrolysis in heterogeneous conditions, the fraction of hydrolyzed glycosidic bonds *S* was estimated for the different hydrolysis times. *S* is related to the number-average degree of polymerization *DP* accordingly to *S* = 1/*DP* − 1/*DP*_0_, where *DP*_0_ is the initial number-average degree of polymerization. The pseudo zero-order kinetics Equation (5) is traditionally used for the hydrolysis of carbohydrates as cellulose [76,77]

(5) $$\frac{1}{DP}-\frac{1}{DP_{0}}=k\cdot t\;(k:\text{apparent rate constant})$$

which is an approximation of the first-order equation [78]

(6) $$S=n^{0}\cdot \left(1-e^{-k\cdot t}\right)$$

where *n*^0^ is the fraction of available glycosidic bonds (accessibility) [79].

Nevertheless, Equation (6) could not be used to model the experimental data of the hydrolysis in heterogeneous conditions. In the literature, Equation (5) appears largely satisfied when *S* correspond to a few units, i.e., *S* << *n*^0^ or *kt* <<1. Thus, Equations (5) and (6) have been successfully used for the carbohydrate degradation in solution, where all glycosidic bonds are available for the degradation [80,81]. In heterogeneous conditions, the carbohydrate hydrolysis kinetics were frequently treated in the same way as in solution, but the use of these equations was debated [82]. In cellulose studies, where the observed downward curvature in the kinetics plots neither followed the zero-order Equation (5), nor the first-order Equation (6) [83], the sum of parallel first-order processes ranked in two or three main groups has been proposed, for which glycosidic bonds degrade approximately at the same rate (Equation (7)) [84]

(7) $$S=n_{w}^{0}\cdot \left(1-e^{-kw\cdot t}\right)+n_{a}^{0}\cdot \left(1-e^{-ka\cdot t}\right)+n_{c}^{0}\cdot \left(1-e^{-kc\cdot t}\right)$$

**Figure 8:**
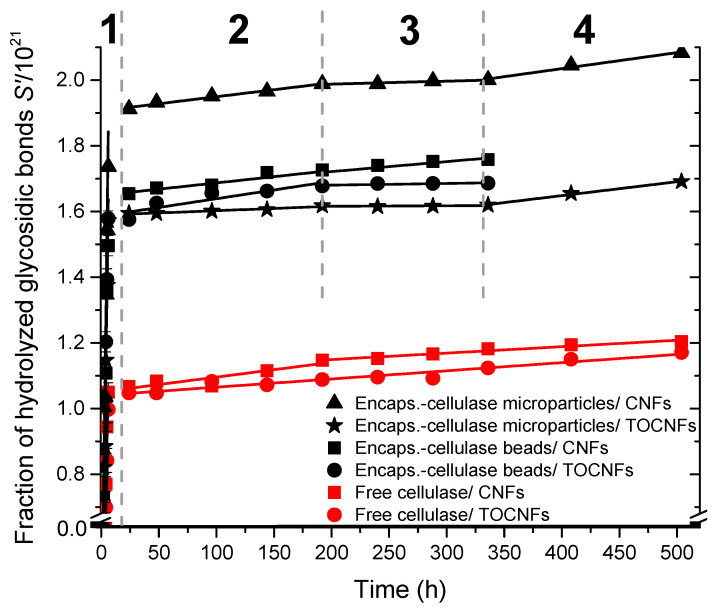
Evolution of the fraction of hydrolyzed glycosidic bonds *S*′ with the hydrolysis time during the enzymatic degradation of cellulose nanofibers CNFs and TOCNFs, by the cellulase immobilized in the alginate beads or microparticles in comparison to the free enzyme; at maximum enzyme activity conditions (pH 4.8, T = 55 °C). With the free cellulase, two degradation regimes (1 and 2) were observed for TOCNFs hydrolysis (-●-) and three regimes (1, 2, 3) for CNFs hydrolysis (-■-). With the cellulase immobilized in the alginate beads, three regimes were observed until 336 h (14 d) (-■-: CNFs; -●-: TOCNFs). With the cellulase immobilized in the alginate microparticles, four regimes (1, 2, 3, and 4) were observed until 504 h (21 d) (-▲-: CNFs; -*-: TOCNFs).

In heterogeneous degradation of cellulose, a global deviation was explained invoking the presence of the so-called weak links (w) or “high defects” [83,85,86]. Two distinct stages during the degradation were hypothesized: a fast initial attack of the weak links followed by a slower degradation of the rest of the amorphous fraction (a) [85]. The presence of weak links was indeed proposed to explain the differences observed on the initial rate of cellulose degradation in heterogeneous conditions [87]. In the view of Equation (7), cellulose was composed of amorphous domains (a) with some weak links (w) and crystalline regions (c) [88]. In our study, we could relate the fraction of hydrolyzed glycosidic bonds *S* (Equation (5)) to the concentration of sugar reducing ends (*RE*) produced during the degradation of the cellulose nanofibers (see Section 2.3) for different hydrolysis times. A fraction of hydrolyzed glycosidic bonds could be expressed

(8) $$\frac{N_{A}\cdot RE}{DP_{0}}-\frac{1}{DP_{0}}=(N_{A}\cdot RE-1)\cdot \frac{1}{DP_{0}}=k\cdot t$$

where *N_A_* is the Avogadro’s number: 6.02 × 10^23^ mol^−1^. Then, it can be written:

(9) $$S^{\prime }=S\cdot DP_{0}=N_{A}\cdot RE-1=DP_{0}\cdot k\cdot t=k^{\prime }t$$

where the fraction of hydrolyzed glycosidic bonds *S* is redefined as *S*′ and *k′ = DP*_0_*.k* represents a pseudo rate constant. In the Figure 8, *S*’ versus time was plotted to study the kinetics of degradation of cellulose nanofibers at 55 °C and pH 4.8. Deviations from Equation (9) are clearly observed in the hydrolysis kinetics plots of cellulose nanofibers, corresponding to heterogeneous conditions. As expected for semi-crystalline cellulose with high *DP*_0_ [88], as present in the cellulose nanofibers, the degradation displays different regimes. The degradation kinetics in Figure 8 shows two, three, or even four distinct regimes. We also used Equation (7) as a guideline for the interpretation of our results. At the earliest times of degradation (until 6 h) with *kt* << 1, the so-called Ekenstam’s approximation could be applied to the ‘weak links’ degradation kinetics. Thus Equation (7) could be re-written as

(10) $$S^{\prime }={k^{\prime }}_{1}\cdot t\;\mathrm{at}\;t<6\;h;$$

These interpretations allowed us to calculate the hydrolysis pseudo rate constants (*k*′_1_) of the weak links from the slopes of the degradation kinetics curves in the first regime (1) below 6 h (Figure 8). After hydrolysis of the weak links in regime 1, we assume that the hydrolysis of the initially less accessible bonds triggers regime 2 (until ca. 192 h) with slower degradation of the remaining amorphous regions (a), characterized by a pseudo rate constant *k*′_2_. Further, the degradation of the crystalline parts should start (regime 3, until ca. 336 h), in which the slowest depolymerization occurs (*k*′_3_) due to topological constraints constituted by crystallites [82]. After a representative hydrolysis of crystals in regime 3, we assume that the parallel hydrolysis of initially less accessible bonds in the amorphous region triggers regime 4, for which the obtained pseudo rate constants *k*′_4_ was similar to the *k*′_2_ estimated in regime 2 (assigned to the degradation of amorphous regions). In Table 2, the pseudo rate constants *k*′_1_, *k*′_2_, *k*′_3_, and *k*′_4_ are summarized, corresponding to the different regimes observed during the enzymatic degradation of cellulose nanofibers. These values were deduced for the free enzyme and the cellulase immobilized in alginate beads or micro-sized particles and for the degradation of the cellulose nanofibers CNFs and TOCNFs.

**Figure 9:**
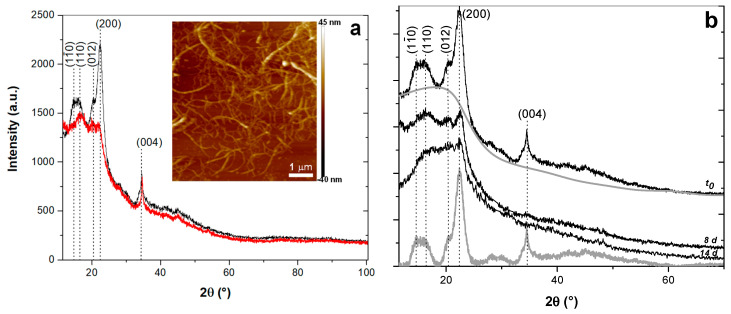
X-ray diffraction (XRD) patterns of: (**a**) starting cellulose nanofibers CNFs (black) and TOCNFs (red); (**b**) CNF after different times of enzymatic degradation with cellulase encapsulated in alginate microparticles. Grey curves in (**b**) show the separated crystalline peaks of the CNF diffractogram obtained by subtracting the amorphous contribution which was estimated by applying a cubic B-spline (also in grey) for curve fitting. The XRD patterns at the different times were vertically shifted for clarity. Inset in (**a**): AFM topography of the CNFs nanofibril network.

Figure 9 shows atomic force microscopy (AFM) images and X-ray diffraction (XRD) patterns of the cellulose nanofiber samples used in this work (CNFs and TOCNFs). AFM images reveal an entangled network of interconnected nanofibrils with average width of 35.2 ± 8.1 nm and bundles up to 100 nm width. Figure 9a shows the XRD patterns of the starting CNFs and TOCNFs. CNFs and TOCNFs displayed diffraction peaks at 2θ = 14.8°, 16.4°, 22.6°, and 34.2° corresponding to the (110), (110), (200), and (040) crystallographic planes of the crystal structure of cellulose I [89]. After the pristine cellulose was oxidized by TEMPO/NaBr/NaClO, no significant changes about the peak position were observed [90,91,92]. Nevertheless, the diffractograms of the starting nanofibers revealed that the TOCNFs were less crystalline than the unmodified CNFs.

The crystallinity index *CrI* of the cellulose nanofibers was calculated from the separated crystalline peak areas of the X-ray diffraction patterns for different times of degradation with the enzyme encapsulated in the alginate particles. A cubic B-spline was used for curve fitting of the amorphous contribution of the diffractograms of the cellulose nanofibers as shown in Figure 9b. Then, the crystalline contribution area (*Area*_Crystalline_) was obtained from the subtraction of this amorphous contribution from the total diffractogram area (*Area*_Total_). This allowed calculating *CrI* by integration of the crystalline peaks and the total diffraction pattern, according to the Equation (11)

(11) $$CrI=\frac{{Area}_{\mathrm{Crystalline}}}{{Area}_{\mathrm{Total}}}\times 100$$

The obtained *CrI* values of the starting cellulose nanofibers and those obtained after different times of enzymatic degradation with the cellulase encapsulated in the alginate microparticles are reported in Table 3. For example, after 336 h (14 d) the *CrI* of the CNFs decreased from 23% to 7.5%. This *CrI* decrease confirms the hydrolysis of crystallinity regions of the cellulose nanofibers during the degradation of these latter by the immobilized cellulase.

## 4. Conclusions

Extrusion-dropping and inkjet-based technologies were successfully used to produce alginate beads and microparticles, respectively, in which the enzyme cellulase could be immobilized to degrade cellulose substrates including crystalline cellulose nanofibers in heterogeneous conditions. The maximum catalytic activity of the enzyme was achieved at pH 4.8 and 55 °C for studies performed with cellulase encapsulated in alginate particles. The optimization of the factors affecting the activity of immobilized cellulose in the hydrolysis of cellulose substrate in solution revealed that the highest enzyme activity with spherical particles could be achieved by dispensing 2% (w/v) alginate into a 0.2 M CaCl_2_ gelation bath and leaving hardening the particles for 1 h. These optimum conditions were considered in the processing of enzyme-entrapped alginate particles for the successful biodegradation of crystalline cellulose nanofibers in suspension. The effect of temperature, pH, and incubation time on the enzymatic activity was investigated. The encapsulated cellulase displayed higher activity compared to the free enzyme over large ranges of temperature and pH and for longer times. The storage stability of immobilized enzymes in comparison to free enzymes demonstrated that the cellulase encapsulation effectively prevented the deactivation of the enzyme. The presented results allowed us to develop a new technological approach to achieve the biodegradation of crystalline nanofibers of both non-modified and TEMPO-oxidized cellulose, looking for an effective application of cellulose nanofibers as reinforcement of biomaterials for tissue engineering.

## Figures and Tables

**Table 1:** Matrix of experimental runs for full factorial design to evaluate the relative activity of immobilized cellulase in relation to sphericity, at pH 4.8 and 60 °C, of the hydrated alginate particles from different formulations obtained by varying the processing parameters. The mean particle sizes of the hydrated and dried alginate beads are also displayed.

	Independent Variables				Responses		Particle Size	
Formu-Lation	c(ALG) (% (w/v))	c(CaCl_2_) (mol/L)	c(NaCl) (mol/L)	Hardening Time (h)	Sphericity Factor	Cellulase Rel. Act. (%)	Hydrated	Dried
							(mm)	
F1	2 (−1)	0.2 (−1)	0 (−1)	1 (−1)	0.004	99.28	2.38 ± 0.20	1.37 ± 0.19
F2	2 (−1)	0.2 (−1)	0 (−1)	3 (1)	0.016	98.91	2.37± 0.25	1.41 ± 0.16
F3	2 (−1)	0.2 (−1)	0.8 (1)	1 (−1)	0.008	90.71	2.29 ± 0.20	1.33 ± 0.23
F4	2 (−1)	0.2 (−1)	0.8 (1)	3 (1)	0.014	90.24	2.06 ± 0.21	1.17 ± 0.18
F5	2 (−1)	0.4 (1)	0 (−1)	1 (−1)	0.011	97.94	2.29 ± 0.21	1.32 ± 0.22
F6	2 (−1)	0.4 (1)	0 (−1)	3 (1)	0.003	96.43	2.00 ± 0.35	1.46 ± 0.25
F7	2 (−1)	0.4 (1)	0.8 (1)	1 (−1)	0.009	93.60	2.19 ± 0.21	1.14 ± 0.16
F8	2 (−1)	0.4 (1)	0.8 (1)	3 (1)	0.012	91.17	2.42 ± 0.21	1.54 ± 0.22
F9	3 (0)	0.2 (−1)	0 (−1)	1 (−1)	0.039	79.86	2.29 ± 0.18	1.37 ± 0.16
F10	3 (0)	0.2 (−1)	0 (−1)	3 (1)	0.054	84.32	2.39 ± 0.21	1.66 ± 0.20
F11	3 (0)	0.2 (−1)	0.8 (1)	1 (−1)	0.031	78.41	2.44 ± 0.20	1.56 ± 0.21
F12	3 (0)	0.2 (−1)	0.8 (1)	3 (1)	0.045	78.41	2.48 ± 0.19	1.43 ± 0.14
F13	3 (0)	0.4 (1)	0 (−1)	1 (−1)	0.041	78.50	2.21 ± 0.16	1.47 ± 0.17
F14	3 (0)	0.4 (1)	0 (−1)	3 (1)	0.031	79.42	2.23 ± 0.14	1.57 ± 0.18
F15	3 (0)	0.4 (1)	0.8 (1)	1 (−1)	0.035	75.44	2.53 ± 0.23	1.42 ± 0.16
F16	3 (0)	0.4 (1)	0.8 (1)	3 (1)	0.041	81.36	2.07 ± 0.18	1.70 ± 0.17
F17	5 (1)	0.2 (−1)	0 (-1)	1 (−1)	0.077	80.18	2.29 ± 0.22	1.55 ± 0.19
F18	5 (1)	0.2 (−1)	0 (−1)	3 (1)	0.065	76.86	2.13 ± 0.57	1.73 ± 0.17
F19	5 (1)	0.2 (−1)	0.8 (1)	1 (−1)	0.087	77.06	2.44 ± 0.14	1.44 ± 0.13
F20	5 (1)	0.2 (−1)	0.8 (1)	3 (1)	0.111	77.21	2.29 ± 0.18	1.43 ± 0.19
F21	5 (1)	0.4 (1)	0 (−1)	1 (−1)	0.068	74.18	2.22 ± 0.17	1.09 ± 0.14
F22	5 (1)	0.4 (1)	0 (−1)	3 (1)	0.069	73.11	2.20 ± 0.17	1.19 ± 0.26
F23	5 (1)	0.4 (1)	0.8 (1)	1 (−1)	0.099	70.19	2.10 ± 0.13	1.19 ± 0.25
F24	5 (1)	0.4 (1)	0.8 (1)	3 (1)	0.086	76.41	2.25 ± 0.13	1.24 ± 0.26
Levels	(−1): 2% (w/v); (0): 3% (w/v); (1): 5% (w/v)	(−1): 0.2 mol/L; (1): 0.4 mol/L	(−1): 0; (1): 0.8 mol/L	(−1): 1 h; (1): 3h				

**Table 2:** Pseudo-rate constants *k*′_1_, *k*′_2_, *k*′_3_, and *k*′_4_ of the kinetics of degradation of cellulose nanofibers CNFs and TOCNFs by the cellulase immobilized in alginate microparticles or beads and by the free enzyme.

Pseudo-Rate Constants/10^14^ (s^−1^)	Immobilized-Cellulase Microparticles		Immobilized-Cellulase Beads		Free Cellulase	
	CNFs	TOCNFs	CNFs	TOCNFs	CNFs	TOCNFs
**at 55 °C, pH 4.8:**						
***k*′_1_**	760 ± 80	690 ± 60	660 ± 20	650 ± 30	350 ± 30	260 ± 30
***k*′_2_**	1.20 ± 0.10	0.38 ± 0.10	1.10 ± 0.20	1.50 ± 0.40	1.30 ± 0.30	0.69 ± 0.06
***k*′_3_**	0.25 ± 0.06	0.042 ± 0.01	0.85 ± 0.10	0.15 ± 0.06	0.54 ± 0.05	-
***k*′_4_**	1.40 ± 0.2	1.2 ± 0.40	*n.d.*	*n.d.*	-	-
**at 37 °C, pH 6.5:**						
***k*′_1_**	*n.d.*	*n.d.*	*n.d.*	*n.d.*	*n.d.*	*n.d.*
***k*′_2_**	2.60 ± 0.70	2.30 ± 0.60	*n.d.*	*n.d.*	1.20 ± 0.30	1.10 ± 0.30
***k*′_3_**	1.60 ± 0.40	1.30 ± 0.50	*n.d.*	*n.d.*	-	-
***k*′_4_**	3.10 ± 0.05	2.70 ±0.30	*n.d.*	*n.d.*	-	-

*n.d.*: non determined.

**Table 3:** Crystallinity index *CrI* of the starting cellulose nanofibers (CNFs) and after different times of enzymatic degradation with cellulose entrapped in alginate microparticles.

Time of Enzymatic Degradation (h)	CrI (%)
0	23
192	9.2
336	7.5

## Supplementary Materials

The following are available online at https://www.mdpi.com/2073-4360/12/7/1522/s1, Figure S1: Size exclusion chromatography SEC/MALLS analysis and FTIR spectroscopy characterization of the starting alginate; Figure S2: Relative activity of cellulase encapsulated in alginate beads in comparison to free enzyme towards degradation of cellulose substrate in solution for 3% and 5% (w/v) alginate formulations (Table 1) at different temperatures, pHs and times; Figure S3: X-ray diffraction patterns and thermogravimetric analysis (TGA) of cellulase/alginate microparticles and beads; Table S1: ANOVA analysis for the enzyme relative activity response surface model; Table S2: ANOVA analysis for the particle sphericity factor (SF) response surface model.
