# Supplementary material for: Biodegradation of Crystalline Cellulose Nanofibers by Means of Enzyme Immobilized-Alginate Beads and Microparticles
Source: Polymers (Basel). 2020 Jul 9;12(7):1522. doi: 10.3390/polym12071522 (PMC7407417; doi:10.3390/polym12071522)
Supplement: Supplementary file 1 [file polymers-12-01522-s001.pdf]

Supplementary Material

Biodegradation of Crystalline Cellulose Nanofibers by Means of Enzyme Immobilized-Alginate Beads and Microparticles

Arnaud Kamdem Tamo [1, 2, 3], Ingo Doench [1, 2, 3], Aliuska Morales Helguera [4], Daniel Hoenders [2, 3, 5], Andreas Walther [2, 3, 5], Anayancy Osorio-Madrazo [1, 2, 3*]

[1] Institute of Microsystems Engineering IMTEK, Laboratory for Sensors, University of Freiburg, 79110 Freiburg, Germany; arnaud.kamdem@imtek.uni-freiburg.de (A.K.T.); ingo.doench@imtek.uni-freiburg.de (I.D.)
[2] Freiburg Materials Research Center FMF, University of Freiburg, 79104 Freiburg, Germany
[3] Freiburg Center for Interactive Materials and Bioinspired Technologies FIT, University of Freiburg, 79110 Freiburg, Germany
[4] Chemical Bioactive Center CBQ, Molecular Simulation and Drug Design Group, Central University of Las Villas, 54830 Santa Clara, Cuba; aliuska@uclv.edu.cu (A.M.H.)
[5] Institute for Macromolecular Chemistry, University of Freiburg, 79104 Freiburg, Germany; daniel.hoenders@makro.uni-freiburg.de (D.H.); andreas.walther@makro.uni-freiburg.de (A.W.)
* Correspondence: anayancy.osorio@imtek.uni-freiburg.de; Tel.: +49-761-203-67363 (A.O-M.)

Alginate macromolecular structure characterization by SEC/MALLS and FTIR

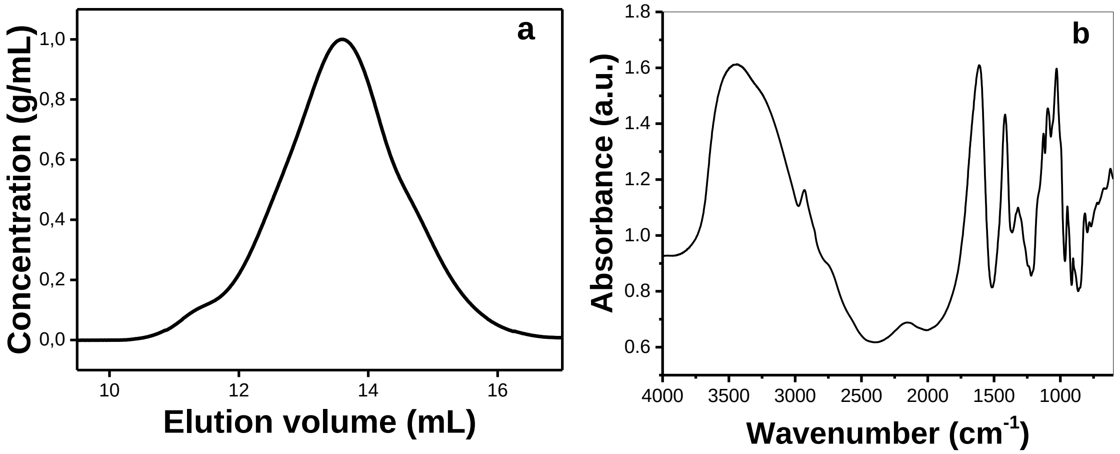

Figure S1: a) SEC/MALLS chromatogram (refractive index detector) of the starting alginate which yielded a weight (Mw) and a number-average molecular weight (Mn) of 9.62×104 (± 0.66 %) and 4.55×104 (± 2.21 %) g/mol, respectively; and a polydispersity index (Mw/Mn) of 2.11 (± 2.30 %), showing a relatively narrow molecular weight distribution. b) FTIR spectrum of the starting alginate, from which a composition of (79 ± 3)% of guluronic acid (G) and of (21 ± 3)% of mannuronic acid (M) was determined; by using the ratio of absorbances of bands at 1320 and 1290 cm-1 (A_{1320}/A_{1290} = 1.18), and at 1125 and 1030 cm^{-1} (A_{1125}/A_{1030} = 0.70).

Activity of encapsulated cellulase towards hydrolysis of cellulose substrate in solution

Figure S2 shows the effect of temperature, pH and time on the activity of cellulase entrapped in alginate beads, obtained by using different formulations containing 3 or 5% (w/v) alginate (see Table 1 in

manuscript), in comparison to free enzyme; during the biodegradation of cellulose substrate (CMC) in solution.

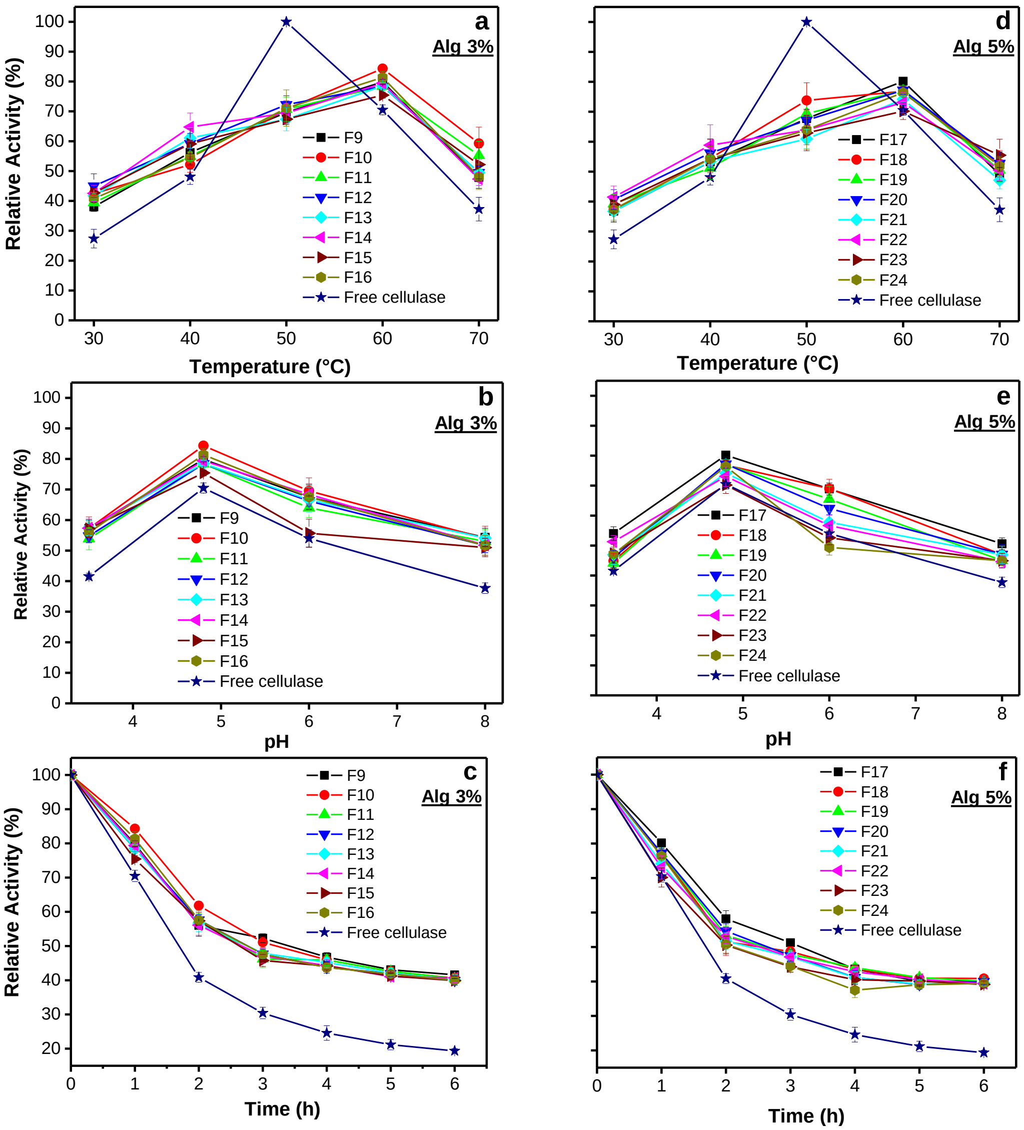

Figure S2: a-c) Relative activity of cellulase encapsulated in alginate beads (see formulation F# description in Table 1 in manuscript) in comparison to that of the free enzyme, showing the degradation of carboxymethylcellulose at: **(a-c)** different temperatures after 1 h at pH 4.8, different pHs after 1 h at 60 °C, and at different times at pH 4.8 and 60 °C for 3% (w/v) alginate formulations; (**d-f)** different temperatures after 1 h at pH 4.8, different pHs after 1 h at 60 °C, and at different times at pH 4.8 and 60 °C for 5% (w/v) alginate formulations.

Optimization for high catalytic activity of the encapsulated cellulase. Multiple Response Optimization

The significance of linear, interaction and quadratic terms of the model we proposed for the simultaneous consideration of multiple responses (section §3.2.4 in the manuscript) was determined using Fisher's statistical test and p-value, as displayed in ANOVA analyses in Tables S1 and S2.

Table S1. ANOVA analysis for the enzyme relative activity response surface model.

	Sum of Square	Degree of Freedom	Mean Square	F-Value	*p*	Comments
(1) c(ALG)	1464.593	1	1464.593	303.3913	< 0.0001	significant
c^2(ALG)	411.189	1	411.189	85.178	< 0.0001	significant
(2) c($CaCl_2$)	28.935	1	28.935	5.9939	0.0307	significant
(3) c(NaCl)	50.896	1	50.896	10.5431	0.0070	significant
(4) HT	3.414	1	3.414	0.7073	0.4167	-
1L by 2L	19.44	1	19.44	4.027	0.0678	-
1L by 3L	28.899	1	28.899	5.9865	0.0307	significant
1L by 4L	0.935	1	0.935	0.1936	0.6677	-
2L by 3L	10.613	1	10.613	2.1986	0.1639	-
2L by 4L	2.407	1	2.407	0.4985	0.4936	-
3L by 4L	4.403	1	4.403	0.9121	0.3583	-
error	57.929	12	4.827	-	-	-
total SS	1854.782	23	-	-	-	-

L : linear

Table S2. ANOVA analysis for the particle sphericity factor (SF) response surface model.

	Sum of Square	Degree of Freedom	Mean Square	F-Value	*p*	Comments
(1) c(ALG)	0.023256	1	0.023256	477.9266	< 0.0001	significant
c^2(ALG)	0.000175	1	0.000175	3.5963	0.082235	
(2) c($CaCl_2$)	0.000108	1	0.000108	2.2250	0.161606	
(3) c(NaCl)	0.000654	1	0.000654	13.4466	0.003225	significant
(4) HT	0.000032	1	0.000032	0.6528	0.434830	
1L by 2L	0.000005	1	0.000005	0.0979	0.759784	
1L by 3L	0.000630	1	0.000630	12.9419	0.003663	significant
1L by 4L	0.000011	1	0.000011	0.2202	0.647311	
2L by 3L	0.000037	1	0.000037	0.7706	0.397254	
2L by 4L	0.000338	1	0.000338	6.9358	0.021831	significant
3L by 4L	0.000038	1	0.000038	0.7706	0.397254	
Error	0.000584	12	0.000049			
Total SS	0.025796	23				

L : linear

Cellulase-immobilized alginate microparticles and beads. Microstructure and thermal properties

The diffractogram of the starting alginate showed two distinct peaks at 2θ of ~13.4° and ~22.9°, corresponding to the (110) and (002) crystallographic planes of the guluronic (G) and mannuronic (M) repeat units (Figure S3-I), which suggests a block copolymer distribution of the G and M units in the polymer chain.[58] The crosslinking of alginate with the Ca^{2+} to produce the Ca-alginate beads resulted in a more crystalline microstructure which diffractogram revealed a relatively sharp peak at 2θ around 15.8° (Figure S3-II). The intensities of the two others peaks originally presented in the sodium alginate were

extremely weak and practically not visible in the X-ray diffraction patterns of the Ca-alginate beads. This could be explained due to the ionic crosslinking interactions which modified the packing structure of the precursory material Na-alginate. X-ray diffraction patterns of particles, in which 0.8 M NaCl was added, also displayed the crystalline peak at 2θ around 15.8° and, as expected, many sharp peaks related to the contribution of inorganic salt (Figure S3-III). In the case of cellulase-immobilized alginate beads, the crystallographic peak at 2θ around 15 was still observed but with significantly less intensity than that observed for the Ca-alginate beads without encapsulated enzyme (Figure S3-IV). This suggests interactions between amino groups of cellulase and carboxylates groups of alginate, possibly disturbing the Ca-alginate crystalline arrangement of the naked particles, as less crystallinity was observed after enzyme addition.

The thermogravimetric analysis (TGA) of the cellulase/alginate particles is shown in Figure 1b. The TGA curves revealed two main thermal degradations at 250 and ~400 °C for the starting sodium alginate, in contrast to the Ca-alginate beads where three main degradations at 210, 300 and ~400 °C were observed. With the enzyme addition into the Ca-alginate, again only two thermal degradations were observed at 270 and 420 °C, shifted to temperatures a little bit higher than those of the sodium alginate. The degradation below 300 °C should correspond to the loss of the alginate hydroxyl groups as water and the degradation of the polymer backbone chains. Above 300 °C, carbonized material slowly decomposes and decarboxylation into CO_2 occurs at the highest temperatures.[59] Besides, at the high degradation temperatures lower weight loss was observed for the enzyme-loaded beads, which thermal stabilization could be due to the formation of polyelectrolyte interactions between alginate polyanions ($–COO^-$) and cellulase polycations ($–NH_3^+$).[58, 60]

Contrary to the diffractogram of the starting sodium alginate showing crystalline peaks, in the Ca-alginate/cellulase microparticles the X-ray scattering patterns just exhibited amorphous halo signals at 2θ around 16 and 43° (in addition to NaCl peak signals when this salt was added) (Figure S3). The thermogravimetric analyses (TGA) of the alginate microparticles showed two main thermal decompositions at 250 and 400 °C, in contrast to three steps observed in the larger beads, which could be related to a multi-structured material allowed to be created during crosslinking of the beads, which formation does not occur during the processing of microparticles.

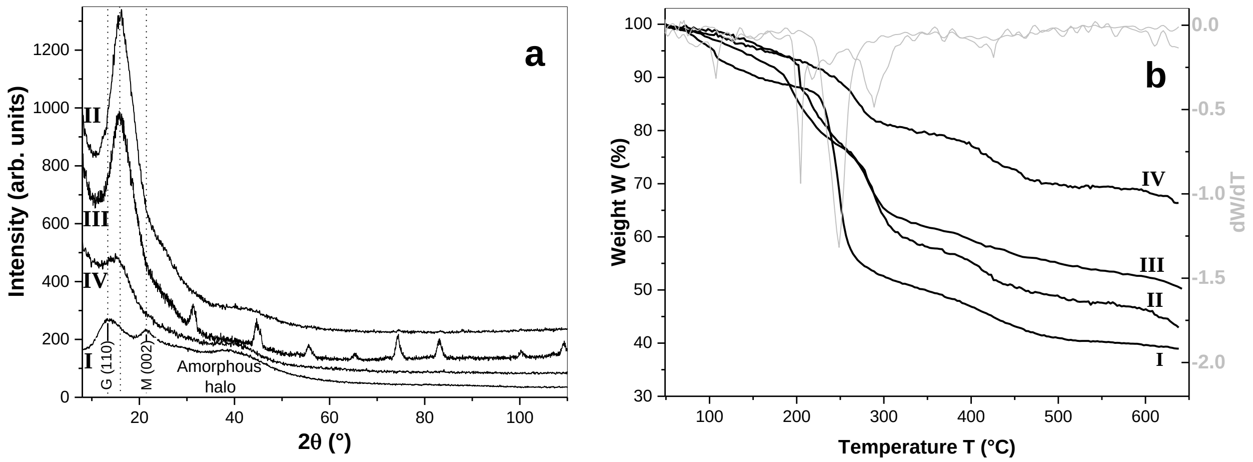

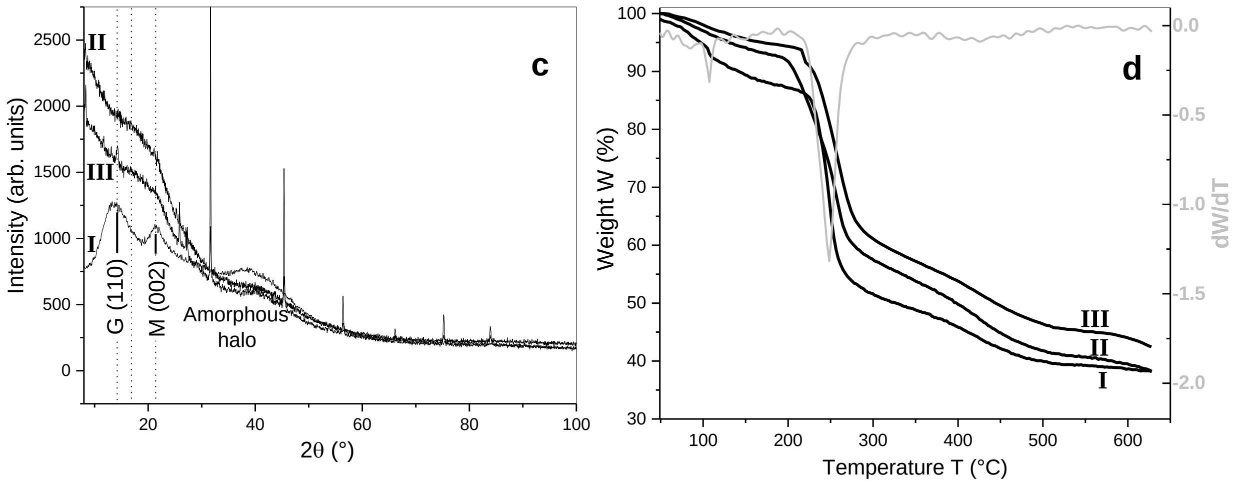

Figure S3. (a) X-ray diffraction (XRD) patterns and (b) thermogravimetric analysis (TGA) of (I) starting sodium alginate powder; dried Ca-alginate capsules obtained by using: (II) 2% (w/v) alginate, 0.2 M $CaCl_2$ alginate bath and 1 h hardening time (formulation F1 in Table 1 of manuscript); (III) 2% (w/v) alginate, 0.2 M $CaCl_2$ alginate bath with addition of 0.8 M NaCl, and 1 h hardening time (formulation F3 in Table 1); (IV) formulation F1 containing immobilized cellulase. (c) XRD patterns and (d) TGA of alginate microparticles obtained by drop-on-demand inkjet technology (4% (w/v) alginate, 0.2 M $CaCl_2$ bath containing 0.2 M NaCl, 1 h hardening time): (II) Ca-alginate microparticles containing cellulase; (III) Ca-alginate microparticles without cellulase.; in comparison to starting sodium alginate (I).
